# The crystal structure of baeocystin

**DOI:** 10.1107/S2056989022004467

**Published:** 2022-05-06

**Authors:** Marilyn Naeem, Alexander M. Sherwood, Andrew R. Chadeayne, James A. Golen, David R. Manke

**Affiliations:** a University of Massachusetts Dartmouth, 285 Old Westport Road, North Dartmouth, MA 02747, USA; b Usona Institute, 2780 Woods Hollow Rd., Madison, WI 53711, USA; c CaaMTech, Inc., 58 East Sunset Way, Suite 209, Issaquah, WA 98027, USA; University of Aberdeen, Scotland

**Keywords:** crystal structure, tryptamines, indoles, hydrogen bonding

## Abstract

The title compound, baeocystin or 4-phosphor­yloxy-*N*-methyl­tryptamine {systematic name: 3-[2-(methylazaniumyl)ethyl]-1*H*-indol-4-yl hydrogen phosphate}, C_11_H_15_N_2_O_4_P, has a single zwitterionic mol­ecule in the asymmetric unit. The mol­ecule has an intra­molecular N—H⋯O hydrogen bond between the ammonium cation and the hydro­phosphate anion. In the crystal, the mol­ecules are linked by N—H⋯O and O—H⋯O hydrogen bonds into a three-dimensional network.

## Abstract 2

The crystal structure of the ‘magic’ mushroom natural product baeocystin is reported for the first time.

## 1. Chemical context

‘Magic’ mushrooms are a group of psilocybin-containing fungi that induce psychoactive effects in humans, and have been used for recreational and sacramental purposes for centuries (Geiger *et al.*, 2018 ▸). Recent studies have shown that psilocybin (4-phosphor­yloxy-*N*,*N*-di­methyl­tryptamine, C_12_H_17_N_2_O_4_P), a naturally occurring tryptamine found in these mushrooms, has great potential in the treatment of mood disorders including anxiety, addiction, depression and post-traumatic stress disorder (Johnson & Griffiths, 2017 ▸; Nutt, 2019 ▸; McClure-Begley & Roth, 2022 ▸). Upon ingestion, psilocybin is converted, *via* hydrolysis of the phosphate ester, to psilocin (4-hy­droxy-*N*,*N*-di­methyl­tryptamine, C_12_H_16_N_2_O), which acts as an agonist of the serotonin (5-hy­droxy­tryptamine or 5-HT) 2A receptor, mediating its psychoactive effects. 
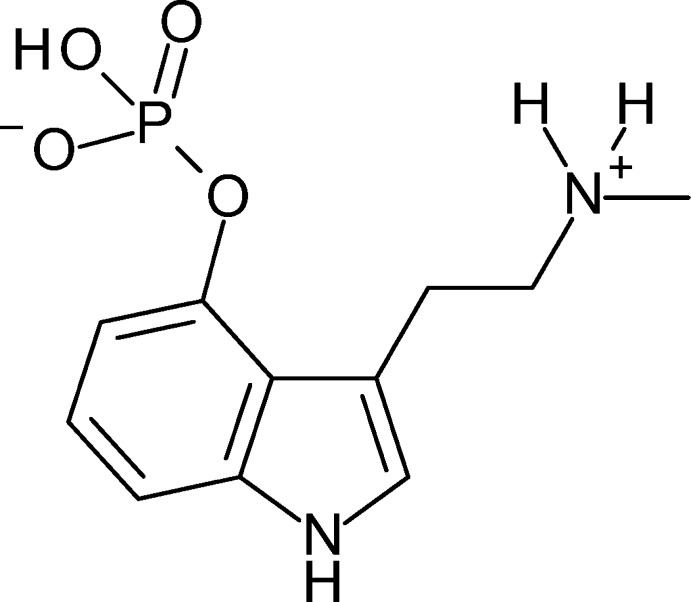


In addition to psilocybin, these mushrooms contain several other structurally similar tryptamines, including norbaeocystin, baeocystin, aeruginascin and norpsilocin. Baeocystin is the *N*-de­methyl­ated analog of psilocybin (4-phosphor­yloxy-*N*-methyl­tryptamine). This minor tryptamine natural product was first isolated from the *Psilocybe baeocystis* mushroom in 1968 (Leung & Paul, 1968 ▸), and has since been found in a number of other mushroom species (Repke *et al.*, 1977 ▸; Gartz, 1987 ▸). The Hoffmeister lab has identified baeocystin as an enzymatic substrate in the synthesis of psilocybin (Fricke *et al.*, 2017 ▸), and also identified norpsilocin (4-hy­droxy-*N*-methyl­tryptamine), the metabolite of baeocystin, as a *Psilocybe* natural product (Lenz *et al.*, 2017 ▸). It was not until 2020 that a scalable synthesis of baeocystin was reported (Sherwood *et al.*, 2020 ▸), with a prior synthesis appearing in the literature in 1988 (Brenneisen *et al.*, 1988 ▸).

Baeocystin’s hydrolysis product and metabolite norpsilocin has been shown to be a full agonist of the 5-HT_2A_ receptor. However, baeocystin does not induce a head-twitch response (HTR) in mice, which is strongly correlated with 5-HT_2A_ receptor-mediated psychoactive effects (Sherwood *et al.*, 2020 ▸). While HTR experiments indicated that baeocystin alone does not induce psychoactive effects, it is still unclear whether it modulates psilocybin’s pharmacology when co-administered. It has been shown that mushroom extracts are an order of magnitude more potent than pure psilocybin in HTR assays (Zhuk *et al.*, 2015 ▸). Additionally, human anecdotal evidence suggests that the experiential psychedelic effects vary between different species of ‘magic’ mushrooms, where the ratios of the different tryptamines can vary significantly.

Our understanding of ‘magic’ mushroom pharmacology has been limited by access to pure, well-characterized chemicals for biological assays. Recent studies have demonstrated the significance of crystallographic characterization of mol­ecules in this area, and in potential pharmaceuticals more broadly (Sherwood *et al.*, 2022 ▸; Toby, 2022 ▸). Herein we report the solid-state structure of the natural product baeocystin, C_11_H_15_N_2_O_4_P, for the first time.

## 2. Structural commentary

**Figure 1:**
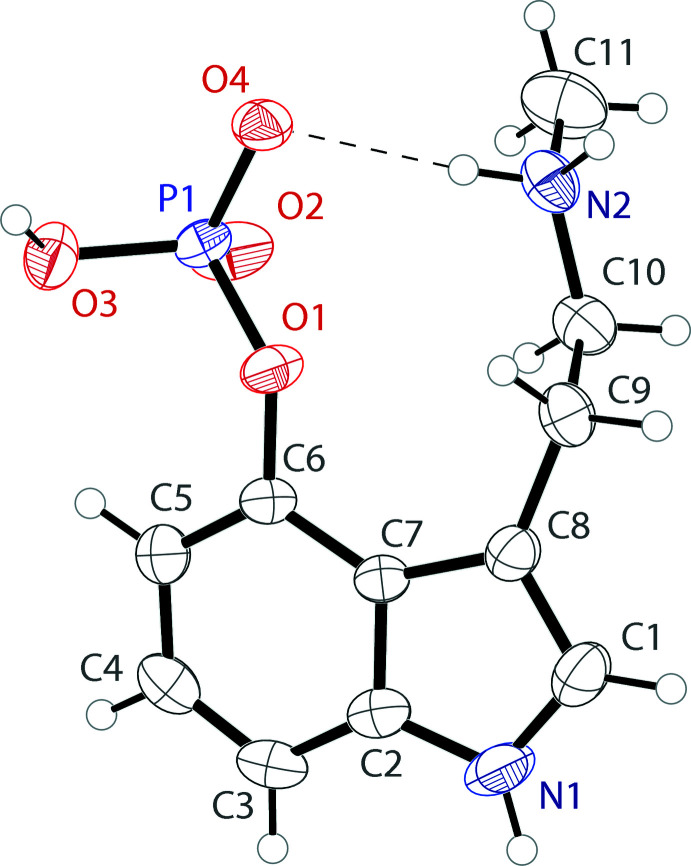
The mol­ecular structure of baeocystin showing the atomic labeling. Displacement ellipsoids are drawn at the 50% probability level. The intra­molecular hydrogen bond is shown as a dashed line.

The asymmetric unit of the baeocystin structure consists of a single zwitterionic tryptamine mol­ecule with a protonated secondary ammonium group and a singly deprotonated phosphor­yloxy unit (Fig. 1 ▸). The phosphate unit shows longer P—O distances with single-bond character for the two-coordinate oxygen atoms, with values of 1.5480 (14) Å for P1—O3 and 1.6032 (12) Å for P1—O1. The bonding about the two one-coordinate oxygen atoms appears to be delocalized, with distances of 1.4848 (14) Å for P1—O2 and 1.5019 (13) Å for P1—O4. The mol­ecule has a near planar indole unit, with an r.m.s. deviation from planarity of 0.016 Å. The ethyl­amino arm is turned away from the indole plane, with a C7—C8—C9—C10 torsion angle of 67.7 (2)° and a C9—C10—N2—C11 unit showing an *anti* conformation with a torsion angle of 178.96 (18)°. The phosphor­yloxy group is similarly turned away from the indole plane, with a C5—C6—O1—P1 torsion angle of 33.8 (3)°. Both groups are turned to the same side of the indole ring, which is likely supported by an intra­molecular N2—H2*A*⋯O4 hydrogen bond (Table 1 ▸).

## 3. Supra­molecular features

**Figure 2:**
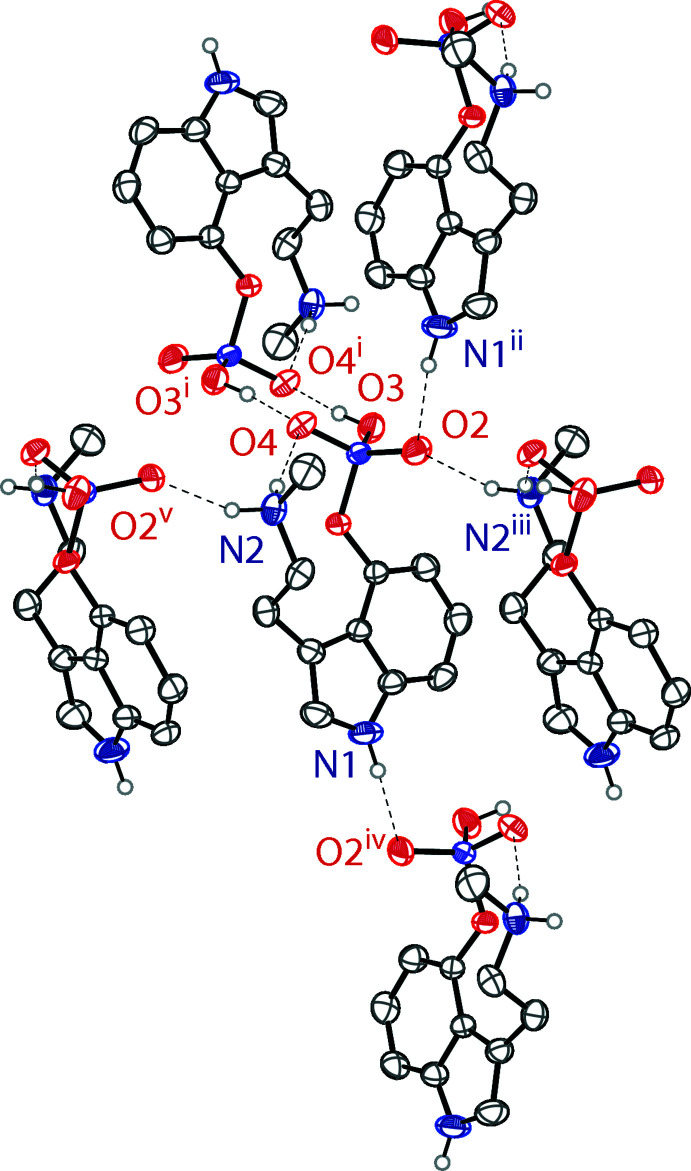
The hydrogen-bonding inter­actions between the baeocystin mol­ecules (Table 1 ▸). Hydrogen bonds are shown as dashed lines. Hydrogen atoms not involved in hydrogen bonding are omitted for clarity. Symmetry codes: (1) 1 − *x*, 1 − *y*, 1 − *z*; (ii) *x*, 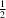 − *y*, 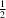 + *z*; (iii) 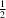 − *x*, − 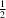 + *y*, *z*; (iv) *x*, 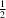 − *y*, − 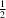 + *z*; (v) 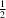 − *x*, 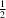 + *y*, *z*.

**Figure 3:**
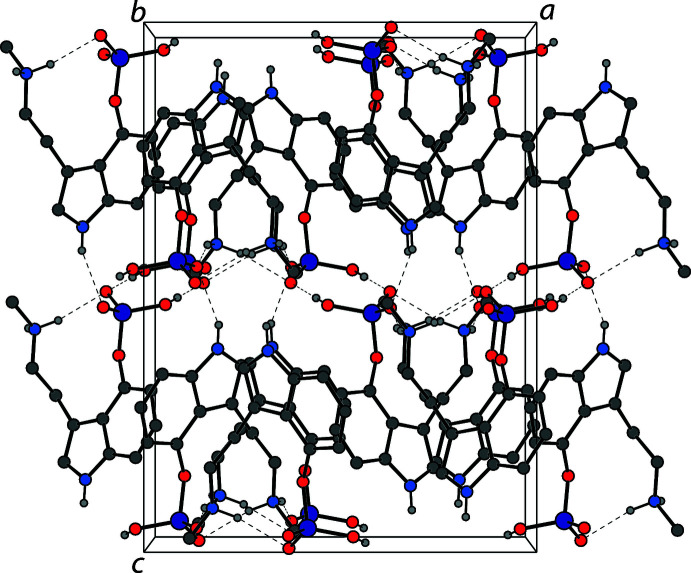
The crystal packing of baeocystin viewed along the *b*-axis direction. Hydrogen bonds are shown as dashed lines. Hydrogen atoms not involved in hydrogen bonds are omitted for clarity.

In the crystal, the baeocystin mol­ecules are held together by various N—H⋯O and O—H⋯O hydrogen bonds that produce a three-dimensional network in the extended structure. The most significant hydrogen bonding observed is the dimerization of two mol­ecules through the phosphate groups, consisting of two O—H⋯O hydrogen bonds. One of the ammonium hydrogen atoms participates in an intra­molecular hydrogen bond as described above, while the other has an inter­molecular N—H⋯O hydrogen bond to a phosphate oxygen atom of a symmetry-generated baeocystin mol­ecule. The indole nitro­gen atom shows an N—H⋯O hydrogen bond to a phosphate oxygen atom of another symmetry-generated baeocystine mol­ecule. One of the phosphate O atoms without a proton is partner in both the intra­molecular N—H⋯O hydrogen bond and the phosphate dimer O—H⋯O hydrogen bond. The other phosphate O atom without a proton is the acceptor to both inter­molecular N—H⋯O hydrogen bonds. Fig. 2 ▸ shows the hydrogen bonding about a single baeocystin mol­ecule, which is also summarized in Table 1 ▸. The crystal packing of baeocystin is shown in Fig. 3 ▸. It is of note that the anhydrate of baeocystin forms from an aqueous solution, while psilocybin readily forms the trihydrate when isolated in a similar fashion. Even the storage of psilocybin anhydrate under humid conditions results in the conversion to the trihydrate, so the ready formation of baeocystin anhydrate is notable (Kuhnert-Brandstätter & Heindl, 1976 ▸).

## 4. Database survey

Perhaps the most closely associated mol­ecule to baeocystin is the well-known psychedelic, psilocybin, whose structure was first reported in 1974 [Weber & Petcher, 1974 ▸: Cambridge Structural Database (CSD; Groom *et al.*, 2016 ▸) refcode PSILOC], and whose crystalline forms have undergone extensive study recently (Sherwood *et al.*, 2022 ▸: TAVZID, TAVZID01; Greenan *et al.*, 2020 ▸: OKOKAD). Similar to baeocystin, psilocybin exists in a zwitterionic form in the solid state. The other closely associated structure to baeocystin is its putative metabolite, norpsilocin, which has been reported as both its free base and its fumarate salt (Chadeayne *et al.*, 2020 ▸: MULXAV, MULXEZ). The only other mono-alkyl­tryptamine structure in the CSD is the free base of 5-meth­oxy-*N*-methyl­tryptamine (Bergin *et al.*, 1968 ▸: QQQAHA) and the only other 4-phospho­ryloxytryptamine structure is of the psilocybin analogue 4-phosphor­yloxy-*N*,*N*-di­ethyl­tryptamine (Baker *et al.*, 1973 ▸: KOWHOT).

## 5. Synthesis and crystallization

Baeocystin was prepared according to the literature procedure (Sherwood *et al.*, 2020 ▸). Single crystals suitable for X-ray diffraction studies were grown by the slow evaporation of an aqueous solution.

## 6. Refinement

Crystal data, data collection and structure refinement details are summarized in Table 2 ▸. Hydrogen atoms H1, H2*A*, H2*B* and H3 were found in a difference-Fourier map and were refined isotropically, using DFIX restraints with an N—H(indole) distance of 0.87 (1) Å, N—H(ammonium) distances of 0.90 (1) Å, and an O—H distance of 0.90 (1) Å. Isotropic displacement parameters were set to 1.2 *U*_eq_ of the parent nitro­gen atoms and 1.5 *U*_eq_ of the parent oxygen atom. All other hydrogen atoms were placed in calculated positions [C—H = 0.93 Å (*sp* ^2^), 0.97 Å (*sp* ^3^)]. Isotropic displacement parameters were set to 1.2 *U*_eq_ of the parent carbon atoms.

## Supplementary Material

Crystal structure: contains datablock(s) I. DOI: 10.1107/S2056989022004467/hb8018sup1.cif

Structure factors: contains datablock(s) I. DOI: 10.1107/S2056989022004467/hb8018Isup2.hkl

CCDC reference: 2169087

Additional supporting information:  crystallographic information; 3D view; checkCIF report

## Figures and Tables

**Table 1:** Hydrogen-bond geometry (Å, °)

*D*—H⋯*A*	*D*—H	H⋯*A*	*D*⋯*A*	*D*—H⋯*A*
N1—H1⋯O2^i^	0.87 (1)	2.16 (1)	2.969 (2)	156 (3)
O3—H3⋯O4^ii^	0.89 (1)	1.67 (1)	2.5560 (18)	173 (3)
N2—H2*A*⋯O4	0.90 (1)	2.04 (1)	2.913 (2)	165 (2)
N2—H2*B*⋯O2^iii^	0.90 (1)	1.85 (1)	2.698 (2)	157 (2)

Symmetry codes: (i) 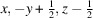 ; (ii) 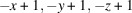 ; (iii) 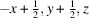 .

**Table 2:** Experimental details

Crystal data	
Chemical formula	C_11_H_15_N_2_O_4_P
*M*_r_	270.22
Crystal system, space group	Orthorhombic, *P* *b* *c* *a*
Temperature (K)	297
*a*, *b*, *c* (Å)	13.229 (1), 10.5551 (7), 17.8346 (13)
*V* (Å^3^)	2490.3 (3)
*Z*	8
Radiation type	Mo *K*α
μ (mm^−1^)	0.23
Crystal size (mm)	0.25 × 0.20 × 0.03

Data collection	
Diffractometer	Bruker D8 Venture CMOS
Absorption correction	Multi-scan (*SADABS*; Bruker, 2018 ▸)
*T*_min_, *T*_max_	0.680, 0.745
No. of measured, independent and observed [*I* > 2σ(*I*)] reflections	58953, 2551, 2155
*R*_int_	0.070
(sin θ/λ)_max_ (Å^−1^)	0.626

Refinement	
*R*[*F* ^2^ > 2σ(*F* ^2^)], *wR*(*F* ^2^), *S*	0.037, 0.096, 1.07
No. of reflections	2551
No. of parameters	180
No. of restraints	4
H-atom treatment	H atoms treated by a mixture of independent and constrained refinement
Δρ_max_, Δρ_min_ (e Å^−3^)	0.21, −0.35

Computer programs: *APEX3* and *SAINT* (Bruker, 2018 ▸), *SHELXT2014* (Sheldrick, 2015*a* ▸), *SHELXL2018* (Sheldrick, 2015*b* ▸), *OLEX2* (Dolomanov *et al.*, 2009 ▸), and *publCIF* (Westrip, 2010 ▸).

## supplementary crystallographic information

### Crystal data

**Table:**

C_11_H_15_N_2_O_4_P	*D*_x_ = 1.441 Mg m^−^^3^
*M**_r_* = 270.22	Mo *K*α radiation, λ = 0.71073 Å
Orthorhombic, *P**b**c**a*	Cell parameters from 9978 reflections
*a* = 13.229 (1) Å	θ = 2.7–26.2°
*b* = 10.5551 (7) Å	µ = 0.23 mm^−^^1^
*c* = 17.8346 (13) Å	*T* = 297 K
*V* = 2490.3 (3) Å^3^	Block, colourless
*Z* = 8	0.25 × 0.20 × 0.03 mm
*F*(000) = 1136	

### Data collection

**Table:**

Bruker D8 Venture CMOS diffractometer	2155 reflections with *I* > 2σ(*I*)
φ and ω scans	*R*_int_ = 0.070
Absorption correction: multi-scan (SADABS; Bruker, 2018)	θ_max_ = 26.4°, θ_min_ = 2.8°
*T*_min_ = 0.680, *T*_max_ = 0.745	*h* = −16→16
58953 measured reflections	*k* = −13→13
2551 independent reflections	*l* = −22→22

### Refinement

**Table:**

Refinement on *F*^2^	4 restraints
Least-squares matrix: full	Hydrogen site location: mixed
*R*[*F*^2^ > 2σ(*F*^2^)] = 0.037	H atoms treated by a mixture of independent and constrained refinement
*wR*(*F*^2^) = 0.096	*w* = 1/[σ^2^(*F*_o_^2^) + (0.0425*P*)^2^ + 1.4406*P*] where *P* = (*F*_o_^2^ + 2*F*_c_^2^)/3
*S* = 1.07	(Δ/σ)_max_ < 0.001
2551 reflections	Δρ_max_ = 0.21 e Å^−^^3^
180 parameters	Δρ_min_ = −0.35 e Å^−^^3^

### Special details

**Table:**

Geometry. All esds (except the esd in the dihedral angle between two l.s. planes) are estimated using the full covariance matrix. The cell esds are taken into account individually in the estimation of esds in distances, angles and torsion angles; correlations between esds in cell parameters are only used when they are defined by crystal symmetry. An approximate (isotropic) treatment of cell esds is used for estimating esds involving l.s. planes.

### Fractional atomic coordinates and isotropic or equivalent isotropic displacement parameters (Å^2^)

**Table:**

	*x*	*y*	*z*	*U*_iso_*/*U*_eq_	
P1	0.41763 (4)	0.35224 (4)	0.45000 (2)	0.02770 (14)	
O1	0.40570 (10)	0.39542 (12)	0.36428 (7)	0.0347 (3)	
O2	0.37058 (12)	0.22578 (13)	0.46057 (8)	0.0471 (4)	
O3	0.53246 (11)	0.34327 (13)	0.46581 (8)	0.0409 (4)	
O4	0.36978 (10)	0.46089 (12)	0.49120 (7)	0.0355 (3)	
N1	0.31807 (15)	0.33361 (18)	0.11856 (9)	0.0481 (5)	
N2	0.17536 (12)	0.48828 (15)	0.41629 (10)	0.0361 (4)	
C1	0.25753 (18)	0.4233 (2)	0.15158 (11)	0.0462 (5)	
H1A	0.206731	0.468024	0.127137	0.055*	
C2	0.38408 (15)	0.28696 (17)	0.17085 (10)	0.0341 (4)	
C3	0.45759 (16)	0.19289 (19)	0.16549 (11)	0.0393 (5)	
H3A	0.469351	0.150336	0.120666	0.047*	
C4	0.51182 (15)	0.16557 (19)	0.22883 (12)	0.0402 (5)	
H4	0.560213	0.101749	0.226826	0.048*	
C5	0.49710 (15)	0.23030 (18)	0.29663 (11)	0.0366 (4)	
H5	0.536699	0.211304	0.338222	0.044*	
C6	0.42355 (14)	0.32217 (16)	0.30106 (9)	0.0281 (4)	
C7	0.36420 (13)	0.35111 (15)	0.23867 (9)	0.0279 (4)	
C8	0.28245 (14)	0.43741 (17)	0.22544 (10)	0.0339 (4)	
C9	0.23077 (15)	0.51840 (18)	0.28322 (11)	0.0376 (4)	
H9A	0.185375	0.577128	0.258397	0.045*	
H9B	0.280986	0.567456	0.310198	0.045*	
C10	0.17092 (15)	0.43738 (18)	0.33870 (11)	0.0393 (5)	
H10A	0.100926	0.433429	0.322673	0.047*	
H10B	0.197693	0.351832	0.338375	0.047*	
C11	0.1188 (2)	0.4085 (2)	0.47055 (14)	0.0580 (6)	
H11A	0.125189	0.443908	0.519921	0.087*	
H11B	0.146014	0.324199	0.470236	0.087*	
H11C	0.048792	0.405930	0.456596	0.087*	
H1	0.315 (2)	0.309 (2)	0.0722 (7)	0.069 (8)*	
H3	0.563 (2)	0.4153 (17)	0.4793 (17)	0.084 (10)*	
H2A	0.2389 (9)	0.491 (2)	0.4340 (12)	0.044 (6)*	
H2B	0.1541 (18)	0.5695 (11)	0.4179 (13)	0.055 (7)*	

### Atomic displacement parameters (Å^2^)

**Table:**

	*U*^11^	*U*^22^	*U*^33^	*U*^12^	*U*^13^	*U*^23^
P1	0.0344 (3)	0.0263 (2)	0.0224 (2)	−0.00431 (18)	−0.00240 (18)	−0.00125 (16)
O1	0.0512 (8)	0.0307 (6)	0.0223 (6)	0.0085 (6)	−0.0036 (5)	−0.0021 (5)
O2	0.0692 (10)	0.0373 (8)	0.0347 (7)	−0.0214 (7)	0.0057 (7)	−0.0022 (6)
O3	0.0394 (8)	0.0343 (7)	0.0491 (8)	0.0059 (6)	−0.0141 (6)	−0.0085 (6)
O4	0.0346 (7)	0.0397 (7)	0.0323 (7)	−0.0039 (6)	0.0015 (6)	−0.0110 (6)
N1	0.0615 (12)	0.0582 (11)	0.0245 (8)	0.0060 (9)	−0.0079 (8)	−0.0055 (8)
N2	0.0324 (9)	0.0300 (8)	0.0459 (10)	0.0012 (7)	0.0046 (7)	−0.0029 (7)
C1	0.0491 (12)	0.0536 (12)	0.0360 (10)	0.0095 (10)	−0.0119 (10)	0.0025 (9)
C2	0.0404 (11)	0.0362 (10)	0.0257 (9)	−0.0040 (8)	0.0026 (8)	−0.0012 (7)
C3	0.0467 (12)	0.0391 (10)	0.0321 (10)	−0.0009 (9)	0.0118 (9)	−0.0078 (8)
C4	0.0379 (10)	0.0366 (10)	0.0461 (12)	0.0079 (8)	0.0092 (9)	−0.0033 (8)
C5	0.0350 (10)	0.0386 (10)	0.0364 (10)	0.0067 (8)	−0.0022 (8)	0.0009 (8)
C6	0.0339 (9)	0.0275 (8)	0.0231 (8)	−0.0001 (7)	0.0023 (7)	−0.0006 (6)
C7	0.0316 (9)	0.0288 (8)	0.0232 (8)	−0.0011 (7)	0.0021 (7)	0.0008 (6)
C8	0.0359 (10)	0.0355 (9)	0.0302 (9)	0.0030 (8)	−0.0032 (7)	0.0017 (7)
C9	0.0350 (10)	0.0331 (9)	0.0447 (11)	0.0060 (8)	0.0007 (8)	0.0011 (8)
C10	0.0352 (10)	0.0369 (10)	0.0458 (11)	−0.0047 (8)	0.0053 (9)	−0.0095 (9)
C11	0.0616 (15)	0.0559 (14)	0.0565 (14)	−0.0113 (12)	0.0208 (12)	0.0013 (12)

### Geometric parameters (Å, º)

**Table:**

P1—O1	1.6032 (12)	C3—H3A	0.9300
P1—O2	1.4848 (14)	C3—C4	1.369 (3)
P1—O3	1.5480 (14)	C4—H4	0.9300
P1—O4	1.5019 (13)	C4—C5	1.402 (3)
O1—C6	1.387 (2)	C5—H5	0.9300
O3—H3	0.893 (10)	C5—C6	1.376 (3)
N1—C1	1.372 (3)	C6—C7	1.396 (2)
N1—C2	1.369 (3)	C7—C8	1.434 (2)
N1—H1	0.866 (10)	C8—C9	1.503 (3)
N2—C10	1.486 (3)	C9—H9A	0.9700
N2—C11	1.485 (3)	C9—H9B	0.9700
N2—H2A	0.899 (10)	C9—C10	1.529 (3)
N2—H2B	0.902 (10)	C10—H10A	0.9700
C1—H1A	0.9300	C10—H10B	0.9700
C1—C8	1.366 (3)	C11—H11A	0.9600
C2—C3	1.393 (3)	C11—H11B	0.9600
C2—C7	1.411 (2)	C11—H11C	0.9600
			
O2—P1—O1	109.59 (8)	C6—C5—C4	119.41 (18)
O2—P1—O3	109.47 (9)	C6—C5—H5	120.3
O2—P1—O4	116.60 (8)	O1—C6—C7	115.48 (15)
O3—P1—O1	106.72 (8)	C5—C6—O1	124.04 (16)
O4—P1—O1	102.00 (7)	C5—C6—C7	120.42 (16)
O4—P1—O3	111.79 (7)	C2—C7—C8	107.72 (16)
C6—O1—P1	126.86 (11)	C6—C7—C2	118.27 (16)
P1—O3—H3	116 (2)	C6—C7—C8	134.01 (16)
C1—N1—H1	125.8 (19)	C1—C8—C7	105.75 (17)
C2—N1—C1	109.14 (16)	C1—C8—C9	127.83 (18)
C2—N1—H1	125.0 (19)	C7—C8—C9	126.27 (16)
C10—N2—H2A	112.1 (15)	C8—C9—H9A	109.4
C10—N2—H2B	111.2 (15)	C8—C9—H9B	109.4
C11—N2—C10	112.48 (16)	C8—C9—C10	111.17 (15)
C11—N2—H2A	105.2 (15)	H9A—C9—H9B	108.0
C11—N2—H2B	111.1 (16)	C10—C9—H9A	109.4
H2A—N2—H2B	104 (2)	C10—C9—H9B	109.4
N1—C1—H1A	124.8	N2—C10—C9	112.34 (16)
C8—C1—N1	110.38 (18)	N2—C10—H10A	109.1
C8—C1—H1A	124.8	N2—C10—H10B	109.1
N1—C2—C3	130.90 (18)	C9—C10—H10A	109.1
N1—C2—C7	107.01 (17)	C9—C10—H10B	109.1
C3—C2—C7	122.07 (17)	H10A—C10—H10B	107.9
C2—C3—H3A	121.3	N2—C11—H11A	109.5
C4—C3—C2	117.34 (17)	N2—C11—H11B	109.5
C4—C3—H3A	121.3	N2—C11—H11C	109.5
C3—C4—H4	118.8	H11A—C11—H11B	109.5
C3—C4—C5	122.42 (18)	H11A—C11—H11C	109.5
C5—C4—H4	118.8	H11B—C11—H11C	109.5
C4—C5—H5	120.3		
			
P1—O1—C6—C5	−33.8 (3)	C2—C3—C4—C5	−1.5 (3)
P1—O1—C6—C7	149.11 (14)	C2—C7—C8—C1	−0.6 (2)
O1—C6—C7—C2	175.07 (15)	C2—C7—C8—C9	175.11 (18)
O1—C6—C7—C8	−4.2 (3)	C3—C2—C7—C6	2.8 (3)
O2—P1—O1—C6	−45.56 (17)	C3—C2—C7—C8	−177.82 (18)
O3—P1—O1—C6	72.88 (16)	C3—C4—C5—C6	2.1 (3)
O4—P1—O1—C6	−169.73 (14)	C4—C5—C6—O1	−177.12 (17)
N1—C1—C8—C7	0.3 (2)	C4—C5—C6—C7	−0.2 (3)
N1—C1—C8—C9	−175.36 (19)	C5—C6—C7—C2	−2.1 (3)
N1—C2—C3—C4	−179.2 (2)	C5—C6—C7—C8	178.63 (19)
N1—C2—C7—C6	−178.69 (17)	C6—C7—C8—C1	178.7 (2)
N1—C2—C7—C8	0.7 (2)	C6—C7—C8—C9	−5.6 (3)
C1—N1—C2—C3	177.8 (2)	C7—C2—C3—C4	−1.0 (3)
C1—N1—C2—C7	−0.6 (2)	C7—C8—C9—C10	−67.7 (2)
C1—C8—C9—C10	107.1 (2)	C8—C9—C10—N2	143.00 (17)
C2—N1—C1—C8	0.2 (3)	C11—N2—C10—C9	−178.96 (18)

### Hydrogen-bond geometry (Å, º)

**Table:**

*D*—H···*A*	*D*—H	H···*A*	*D*···*A*	*D*—H···*A*
N1—H1···O2^i^	0.87 (1)	2.15 (1)	2.969 (2)	156 (3)
O3—H3···O4^ii^	0.89 (1)	1.67 (1)	2.5560 (18)	173 (3)
N2—H2*A*···O4	0.90 (1)	2.04 (1)	2.913 (2)	165 (2)
N2—H2*B*···O2^iii^	0.90 (1)	1.85 (1)	2.698 (2)	157 (2)

Symmetry codes: (i) *x*, −*y*+1/2, *z*−1/2; (ii) −*x*+1, −*y*+1, −*z*+1; (iii) −*x*+1/2, *y*+1/2, *z*.
